# Refractive errors and visual impairment among children and adolescents in southernmost China

**DOI:** 10.1186/s12886-021-01993-5

**Published:** 2021-05-20

**Authors:** Li Peng, Ling Gao, Yunyan Zheng, Yanan Dai, Qing Xie

**Affiliations:** 1 Department of Ophthalmology, Central South University Xiangya School of Medicine Affiliated Haikou Hospital, Haikou, 570208 Hainan China; 2 Department of Ophthalmology, The Second Xiangya Hospital, Central South University, Changsha, 410000 Hunan China; 3 Department of Neurosurgery, Central South University Xiangya School of Medicine Affiliated Haikou Hospital, Haikou, 570208 Hainan China; 4 Department of Ophthalmology, Changsha Central Hospital, University of South China, Changsha, 410004 Hunan China

**Keywords:** Refractive errors, Myopia, Hyperopia, Visual impairment, Children and adolescents, Tropical island

## Abstract

**Background:**

Refractive errors and visual impairment in southernmost China have not been reported previously. We aim to investigate and determine the age-specific prevalence of myopia, hyperopia, astigmatism, and visual impairment based on a large population cross-sectional study in Hainan area of southernmost tropical province in China.

**Methods:**

A population-based sample of 31,524 children aged 615years from Hainan was assessed. Non-cycloplegic autorefraction and visual acuity (VA) analyses were performed on all participants and a subgroup of participants undergoing cycloplegia.

**Results:**

Of all participants, 23.0% presented uncorrected VA (UCVA) was worse than 20/40 in worse eye, 6.0% presented mild presenting visual acuity impairment (PVAI), 7.0% presented moderate PVAI, 0.2% presented severe PVAI in the better eye, and 46.9% presented abnormal UCVA [worse than 20/25 (6,<8years old) and worse than 20/20 (8years and older)] at least in one eye. The overall prevalence of myopia [spherical equivalent (SE)0.50 D] and high myopia (SE6.00 D) were 46.0%, 1.0% respectively. Hyperopia [SE+1.00 D (7years old) and SE+2.00 D (6,<7years old)] and significant hyperopia (SE+3.00 D) were 4.2 and 0.6%, respectively. Astigmatism [cylinder1.00 D (7years old) and1.75 D (6,<7years old)] was found in 31.9%.

**Conclusions:**

Myopia was the most common refractive error in southernmost province in China (Hainan). Its prevalence increased with age, while hyperopia prevalence showed a decreasing trend. However, myopia, especially high myopia prevalence was much lower than in other urban regions across China.

## Background

Refractive errors (RE) are the most common cause of visual impairment and disability in children and adolescents worldwide [1]. They can be classified into myopia, hyperopia and astigmatism [2]. Globally, it was estimated that 12.8 million children have visually impairment from uncorrected refractive errors from 2004, and this is set to rise with the increasing incidence of myopia [3]. Refractive errors represent an excessive increase in myopia which has huge social, educational, and economic consequences to society, especially for those suffering from high myopia [4, 5]. Currently, the prevalence of myopia is increasing worldwide [6]. As is well-known, the highest rates of myopia occur in China with over 80% of the younger generation impacted at present [7], making it a major current concern.

Studies have been performed to excavate the possible factors related to refractive errors [8]. Environmental and genetic factors have both been implicated [8, 9]. Some researchers confirmed that time spent outdoors playing is an important environmental factor for preventing myopia [10]. Previous studies have suggested that daylight exposure holds a doseresponse relationship with ocular axial elongation, which plays a vital role in reducing myopia [11]. Hainan, the only tropical island and province in China, stretches from 3.30N~20.07N latitude with the longest daylight exposure in China. The analysis of visual refractive errors in this isolated island has barely been reported to date. Therefore, it is important to identify the prevalence in Hainan children and adolescents. The purpose of this study is to evaluate the prevalence of myopia, hyperopia, astigmatism and visual impairment in the only tropical region in China. It aims to contribute new data on refractive errors and visual acuity for this population.

## Methods

### Study population

This large population cross-sectional study was approved by the research ethics committees of Central South University Xiangya School of Medicine, Affiliated Haikou Hospital and adhered to the tenets of the Declaration of Helsinki. Written informed consent and assent were obtained from their parent or legal guardian of all participants. Prior to enrolling school children and adolescents, the purpose and methods of this study were explicated by the investigators. This was a cross-sectional study conducted from May 2018 to July 2018 in Haikou, a provincial capital of about 2.2 million people in Hainan province, P. R. China, and it stretches from 19.32N-20.05N latitude with the long daylight exposure in China.

Using randomized method, MeiLan District was randomly selected from 4 districts in the Haikou city, 12 primary schools were selected from 30 primary schools and 7 junior middle schools were selected from 16 junior middle schools. All students from the selected schools were invited to participate. In this study children and adolescents stands for boys and girls aged between 6 and 15years old. The inclusion criteria for our study were as follows: (1) age between 6 and 15years; (2) with no congenital abnormalities; (3) Chinese Han Nationality Students; (4) an informed consent was obtained by the parents or legal guardian. The reasons for exclusion from the analysis of refraction were failure to complete the examination, previous ophthalmic surgery, wearing intraocular lenses, ocular diseases (ocular trauma, cataracts, glaucoma, optic neuropathy) or ocular injuries caused the significant refractive error. Of 31,780 eligible students, 31,524 students took part in this study (99.20% participation rate).

All the participants accepted the UCVA testing, refractive error measurement, slit lamp microscope examination, cover-uncover test, alternate cover test and pupil fundus examination. Before test of visual acuity, students were asked to take off glasses or contact lens. Uncorrected visual acuity (UCVA) was tested for each eye by trained doctors under bright daylight with a measured distance of five meters, by using the standard Logarithmic visual acuity E chart. The values were converted to Snellen for subsequent analyses. The children and adolescents underwent all examinations by two trained ophthalmologists and auto-refractometry was conducted by two experienced senior optometrists. Refractive error (RE) of each eye was measured without cycloplegia at least three times via performing an automatic refractometer (NIDEK, ARK-1, Japan). Three readings of refractive error were taken from each eye and their average was entered for analysis. Repeated measurements were performed if one measured outcome deviated from the other two by more than 0.50 D.

In addition, for better quality control, the professional engineers calibrated the autorefractors every day to minimize the measuring refractive error. About 5% of the students were randomly selected to perform the repeated test of refraction. Data Recording were double entered into Microsoft Excel spreadsheets, meanwhile, by checking the original paper records against the database was conducted to resolve the differences.

### Population subgroup for a cycloplegic autorefraction study

The refraction results were considered to be influenced by active accommodation responses in children with non-cycloplegic autorefraction [1]. A small fraction of these participants with accepting parents were assigned to cycloplegia measurement before and after cycloplegia in a pilot study. Cycloplegia assessment was performed using 1% cyclopentolate eye drops. Refractive error measurements were performed with same protocol as above before and after cycloplegia. A total of 1,006 students participated in the subgroup for a cycloplegic autorefraction study, of these 546 were boys and 457 girls.

### Definitions of myopia, hyperopia and astigmatism

Spherical equivalent refractive errors were counted as the sphere power plus 1/2 of the cylinder power (SER=sphere+cylinder). Considering the non-cycloplegic autorefractor was applied to measure the refractive error, which tended to over-measure the myopic magnitude, especially in in young children, we defined myopia using combination of spherical equivalent and UCVA. Myopia was defined as spherical equivalent (SE)0.50 diopter (D) and UCVA 20/25 or worse in at least one eye. High myopia was defined as spherical equivalent-6.0 diopters (D) and UCVA 20/25 or worse. It was found that the UCVA was taken into consideration can improve the accuracy of myopia using non-cycloplegic autorefractor refractive error. Non-myopia was defined as follows, SE between -0.50 to+1.00D and no glasses or ophthalmic history [12]. Since UCVA was a study factor to consider in all children**,** UCVA 20/20 in both eyes was not used for study exclusion**.** Hyperopia was defined as SE+1.00 D (7years old) and SE+2.00 D (6,<7years old) in at least one eye for the primary analysis. Cylindrical refractive error was classified as positive correcting cylinder form. Astigmatism was defined as absolute values of cylindrical refractive error in at least one eye:1.00D (7years old) and1.75 D (6,<7years old). When one eye was myopic and the other hyperopic, the participant was considered both as a myope and as a hyperope. If a refractive error was present only in one eye, the participant was still categorized into the appropriate eye group for each condition in the analysis. Meanwhile, hyperopia (SE+3.00D) were also calculated and presented in this study for further research.

### Definition of abnormal UCVA

Abnormal UCVA was defined as UCVA worse than 20/25 (6,<8years old) and worse than 20/20 (8years and older) in at least one eye for its clinical diagnosis. Conversely, in all other conditions it was defined as normal UCVA.

### Definition of presenting visual acuity impairment

Based on standard WHO definition, visual impairment reported as presenting visual acuity (PVA). The PVA was considered the uncorrected VA for subjects who do not have corrective eyeglasses. Presenting visual acuity impairment (PVAI) was classified as mild visual impairment, moderate visual impairment and severe visual impairment. Mild visual impairment, Moderate visual impairment and severe visual impairment were defined as presenting visual acuity between 6/12 to 6/18, 6/18 to 6/60, and 6/60 to 3/60 in the better eye.

### Statistical analysis

Prevalence was calculated as the percentage of participants with the particular type of refractive error to the total number of children who successfully completed refraction for at least one eye. Results are showed for 3 age groups from 6 to 15years old. R statistical analysis package (version 3.5.3) was used to statistically analyze the data with a 0.05 significance level for probability (p). Confidence intervals (CI) presented for proportions are exact binomial 95% confidence intervals. No missing data were found.

## Results

### Population characteristics

**Fig. 1:**
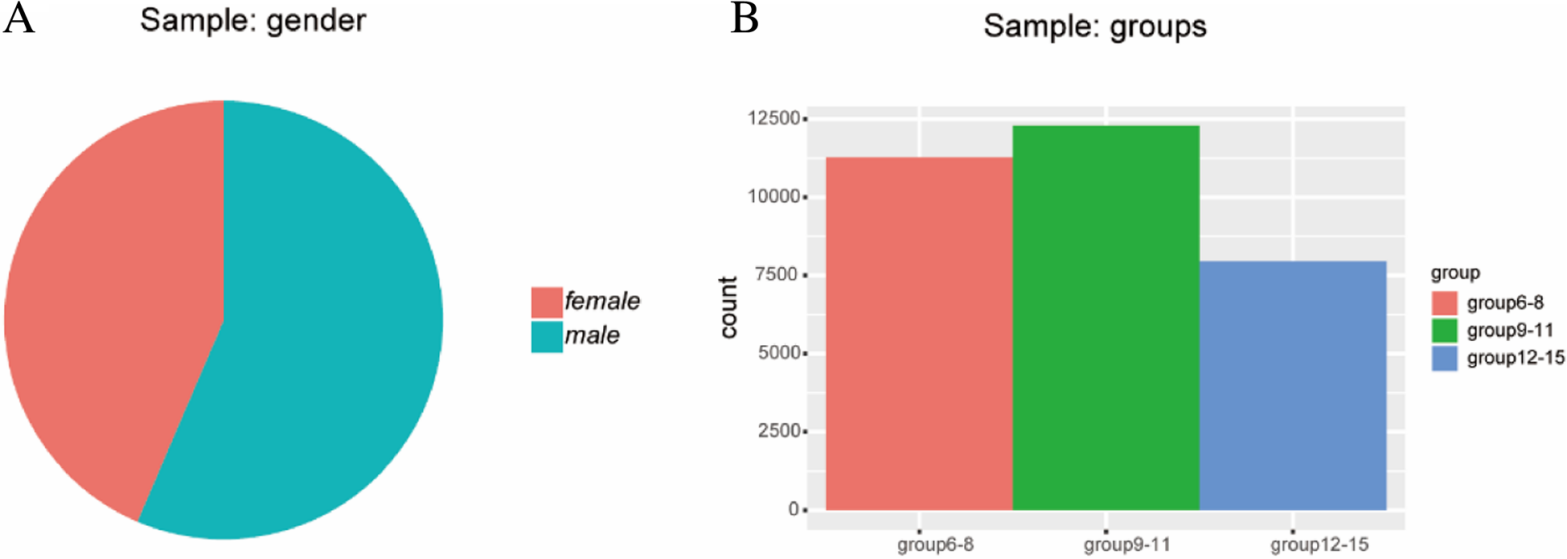
Distribution of sample by gender **a** and age group **b**. The proportion of male participants was a little higher than that of female participants (56.4% vs.43.6%, *P*<0.0001)

Of 31,780 eligible students, 31,524 children and adolescents participated in this study and completed all the clinical eye examinations (99.2% participation rate). 17,794 were boys and 13,730 were girls. The proportion of male participants was a little higher than that of female participants (56.4% vs.43.6%, *P*<0.0001). Mean age of the participants was 9.732.42years (range 6 to 15years). The participants were grouped into three according to their ages (6,<9years old for group6-8;9,<12years old years for group9-11;12; and15years old for group12-15). The number of children were 11,277, 12,292, and 7,955 for the three groups, respectively. The demographic characteristics of the participants are demonstrated in Fig.1a, b.

### Abnormal UCVA

**Fig. 2:**
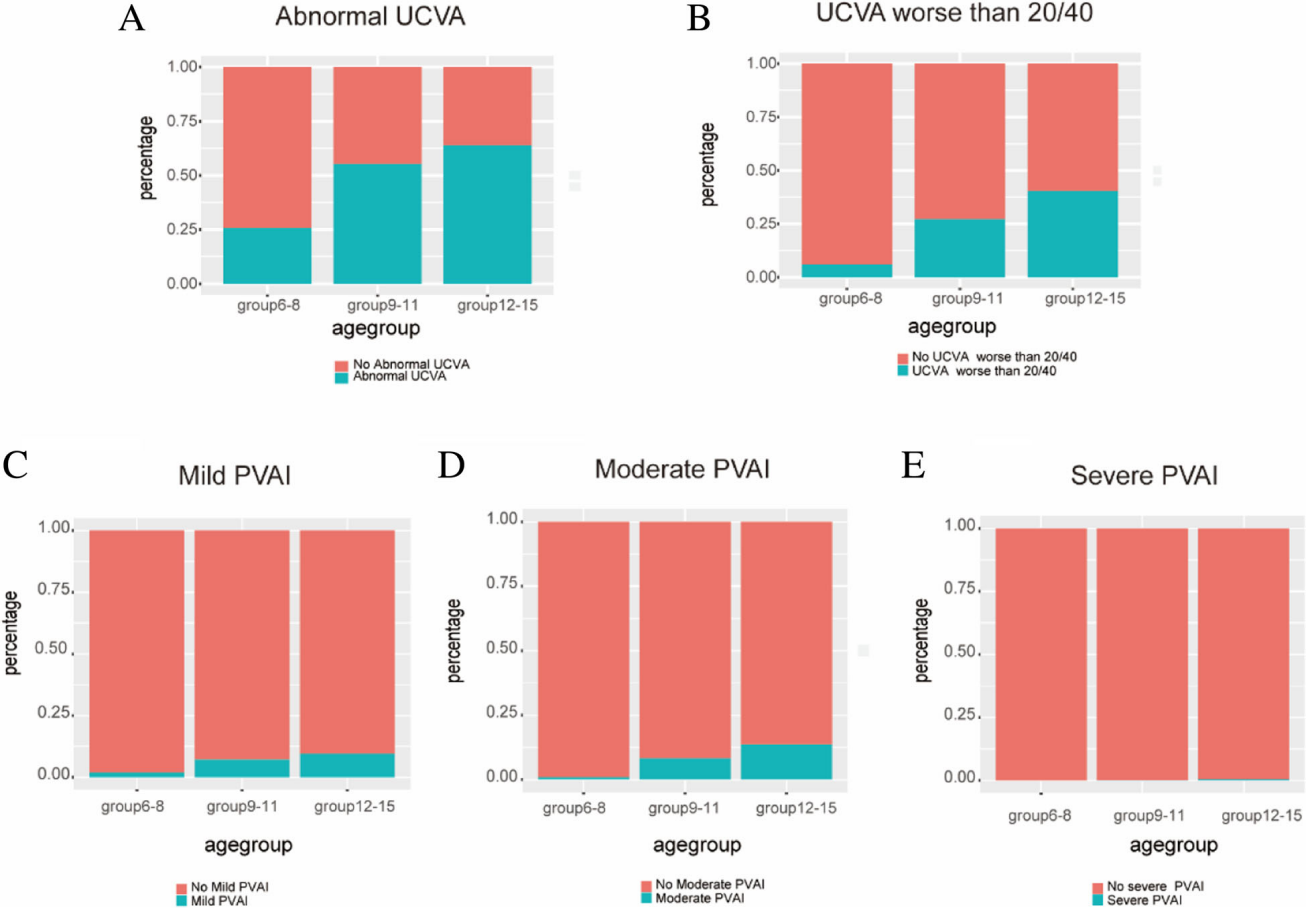
Distribution map of three visual acuity groups. Abnormal UCVA: worse than 20/25 (6,<8years old) and worse than 20/20 (8years and older) at least in one eye **a**. UCVA worse than 20/40 **b**. Mild PVAI **c**. Moderate PVAI **d**. Severe PVAI **e**

Abnormal UCVA was observed in 46.9% (CI: 46.447.5%) of all the participants (14,799/31,524), 25.7% (CI: 24.926.5%) in group6-8 (2,901/11,277), 55.4%(CI: 54.556.2%) of group9-11 (6,806/12,292) and 64.0% (CI: 63.065.1%) of group12-15 (5,092/7,955) (Table 1 and Fig.2a).

**Table 1:** Table of presenting visual acuity. Mean SE (standard deviation, SD) in diopters [D]

Age groups	n	Mild PVAI (CI) (%)	Moderate PVAI (CI) (%)	Severe PVAI (CI) (%)	Abnormal UCVA (CI) (%)	UCVA worse than 20/40 in worse eye (CI) (%)	Mean SE[D]
68	11,277	2.0 (0.180.2)	0.9 (0.71.1)	20.0 (0.00.0)	5.7 (24.926.5)	6.0 (5.66.4)	-0.981.37
911	12,292	7.2 (6.87.7)	8.3 (7.88.8)	0.2 (0.10.2)	55.4 (54.556.2)	27.2 (26.528.0)	-1.161.42
1215	7955	9.6 (CI: 8.910.2)	13.7 (13.014.5)	0.4 (0.30.6)	64.0 (63.065.1)	40.5 (39.441.6)	-1.391.50
Total	31,524	6.0 (5.76.2)	7.0 (6.87.3)	0.2 (0.10.2)	46.9 (46.447.5)	23.0 (22.523.5)	-1.161.43

### Visual acuity impairment

About 23.0% (CI: 22.523.5%) of all participants had UCVA worse than 20/40 in worse eye (7246/31,524), 6.0% (CI:5.66.4%) in group6-8(677/11,277), 27.2% (CI: 26.528.0%) of group9-11(3349/12,292) and 40.5% (CI:39.441.6%) in group12-15 (3,220/7955) (Fig.2b). Differences were seen between groups. The results showed a trend of worsening UCVA with age.

Mild presenting visual acuity impairment (PVAI) was seen in 6.0% (CI: 5.76.2%) of all the participants in the better eye, 2.0% (CI: 0.180.23%) in group6-8, 7.2% (CI: 6.87.7%) in group9-11, 9.6% (CI: 8.910.2%) in group12-15, respectively. Moderate PVAI was seen in 7.0% (CI: 6.87.3%) of all the participants in the better eye, 0.9% (CI: 0.71.1%) in group6-8, 8.3% (CI: 7.88.8%) in group9-11, 13.7% (CI: 13.014.5%) in group12-15, respectively. Severe PVAI was seen in 0.2% (CI: 0.10.2%) of all the participants in the better eye, 0.0% (CI: 0.00.0%) in group6-8, 0.2% (CI: 0.10.2%) in group9-11, 0.4% (CI: 0.30.6%) in group12-15, respectively (Table 1 and Fig.2c, d, e).

### Refractive error varies with age

**Fig. 3:**
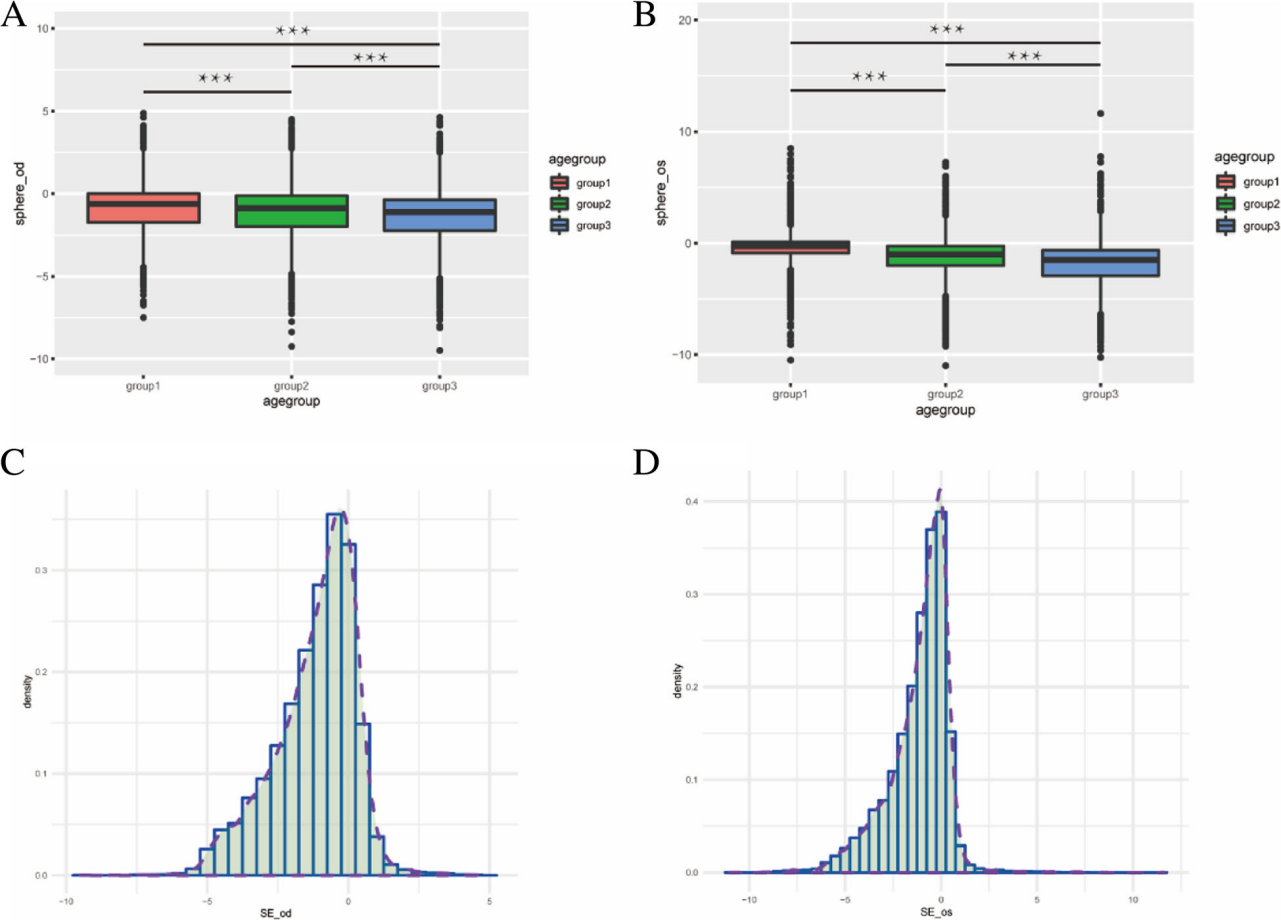
Distribution map of mean SE in the right **a**, **c** and left eyes **b**, **d**

The overall mean SE was -1.16D (1.43), for right eyes and -1.13D (1.56) for left eyes. The mean SE was -0.98 D (1.37) in group6-8, -1.16 D (1.42) in group9-11, and -1.39D (1.50) in group12-15 for the right eyes. The mean SE differences between the three age groups were statistically significant(p1,2,3<0.001), which indicated negative SE increase with age (Table 1 and Fig.3).

### Prevalence of myopia, hyperopia and astigmatism

Table 2 summarizes the prevalence of myopia, hyperopia, and astigmatism determined in each age group between 6 to 15years. The overall prevalence rates of myopia and hyperopia were 46.0% (CI: 45.446.5%) and 4.2% (CI:4.04.4%). Moreover, myopia and hyperopia prevalence rates were 31.2% (CI: 30.432.1%) and 5.6% (CI: 5.26.0%) in group6-8, 50.1% (CI: 49.251.0%) and 3.6% (CI: 3.33.9%) in group9-11, 60.5% (CI:59.561.6%) and 3.2% (CI:3.44.3%) in group12-15, respectively. Differences were seen in myopia and hyperopia prevalence between groups. Both were associated with age. The overall prevalence of high myopia was 1.0% (CI: 0.91.1%); 0.2% (CI: 0.10.3%) in group6-8, 1.0% (CI: 0.81.1%) in group9-11 and 2.1% (CI: 1.82.4%) in group12-15. The overall prevalence of significant high hyperopia (SE+3.00 D) was 0.6% (CI: 0.50.7%); 0.7% (CI: 0.50.8%) in group6-8, 0.6% (CI: 0.50.7%) in group9-11 and 0.6% (CI: 0.40.7%) in group12-15. The overall prevalence of astigmatism was 31.9% (CI: 31.432.4%)**;** 29.9% (CI: 29.030.7%) in group6-8, 32.7% (CI: 31.933.5%) in group9-11 and 33.4% (CI: 32.434.4%) in group12-15.

**Table 2:** Prevalence of myopia, hyperopia, astigmatism and the distribution of different SE

Age groups		n	Myopia % (CI) (SE-0.50D)	Myopia %(CI) (SE-6.00D)	Hyperopia%(CI) (CI) (SE*)	Hyperopia%(CI) (SE+3.00 D)	Stigmatism%(CI) (Cyl*)
68	All	11,277	31.2 (30.432.1)	0.2 (0.10.3)	5.6 (5.26.0)	0.6 (0.50.8)	29.9 (29.030.7)
	Femal	5078	32.7 (31.434.0)	0.2 (0.10.3)	5.6 (4.96.2)	0.7 (0.40.9)	28.6 (27.429.8)
	Male	6199	30.0 (28.931.1)	0.2 (0.10.3)	5.6 (5.16.2)	0.7 (0.50.9)	30.9 (29.832.1)
911	All	12,292	50.1 (49.251.0)	0.93 (0.81.1)	3.6 (3.33.9)	0.6 (0.50.7)	32.7 (31.933.5)
	Femal	5366	55.1 (53.856.4)	1.1 (0.81.4)	3.4 (2.93.8)	0.6 (0.40.8)	31.8 (30.633.1)
	Male	6926	46.2 (45.047.4)	0.8 (0.61.0)	3.8 (3.44.3)	0.6 (0.40.8)	33.4 (32.334.5)
1215	All	7955	60.5 (59.561.6)	2.10 (1.82.4)	3.2 (2.83.6)	0.6 (0.40.7)	33.4 (32.434.4)
	Femal	3286	69.1 (67.670.7)	2.7 (2.23.3)	5.4 (3.14.4)	0.8 (0.51.1)	32.9 (31.334.5)
	Male	4669	54.4 (53.155.9)	1.7 (1.32.0)	2.8 (2.33.3)	0.5 (0.30.6)	33.8 (32.435.1)
Total		31,524	46.0 (45.446.5)	1.0 (0.91.1)	4.2 (4.04.4)	0.6 (0.50.7)	31.9 (31.432.4)

SE*:SE+1.00 D (7years old) and SE+2.00 D (6,<7years old)

Cyl*:Cylinder1.00D(7years old) and+1.75 D(6,<7years old)

**Fig. 4:**
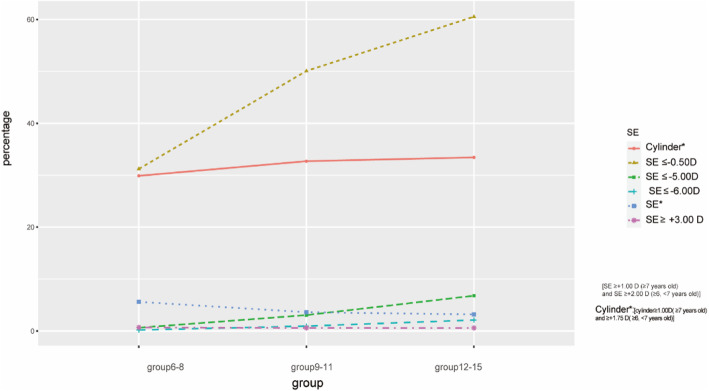
Changes of distribution lines of myopia, hyperopia, astigmatism and the distribution of different SE with age groups. Locally weighted regression lines derived for prevalence of myopia (SE-0.50D,-5.00D,-6.00D), hyperopia (SE*), Significant hyperopia (SE+3.00 D) and astigmatism (cylinder*) as a function of age group for Hainan children and adolescents

Figure4 illustrates the prevalence of myopia, hyperopia and astigmatism by year of age in 6 to 15-year-olds. Myopia and high myopia prevalence appeared increased across the age range with a significant trend, but this contrasted with hyperopia prevalence. Results for astigmatism revealed that prevalence appeared relatively stable with no significant trend**.** Different SE statuses were also calculated (Table 2, Fig.4).

### Refractive errors prevalence

Refractive errors include myopia, hyperopia and astigmatism. If at least one of three conditions was met, then participants were categorized as refractive errors. The overall prevalence rates of prevalence of refractive errors was 59.6% (CI:59.060.1%).

### Cycloplegic autorefraction subgroup study results

About 1,003 children and adolescents included in the subgroup of participants 546 were boys and 457 were girls with a mean age of 9.902.30. (median:10years; range: 615years). The overall mean SE was -1.271.43D, -0.901.66 D for right eyes and -1.241.5D, -0.841.75D for left eyes before and after cycloplegia respectively. The average difference of SE was -0.370.85D for right eyes and -0.400.84 D for left eyes before and after cycloplegia.

## Discussion

Based on the data from large population-based multi-age group studies, we presented prevalence estimations for myopia, hyperopia and astigmatism in Hainan, the tropical island of China with children aged 6 to 15years old. This is the first report about refractive errors in this emblematic population of children and adolescents in farthest south China. It has revealed myopia to be the most common type of refractive error.

In our study, we found that prevalence of myopia, high myopia and uncorrected visual acuity impairment was lower in the first 3years of primary school, but increased with age and grade as intensive continuing education increased. However, hyperopia prevalence declined with age. The average SE results were -0.98 D (1.37), -1.16 D (1.42), and -1.39D (1.50) in the above three age groups respectively. In addition, low myopia is the most common form of myopia, but the prevalence of high myopia increased with advancing age.

**Fig. 5:**
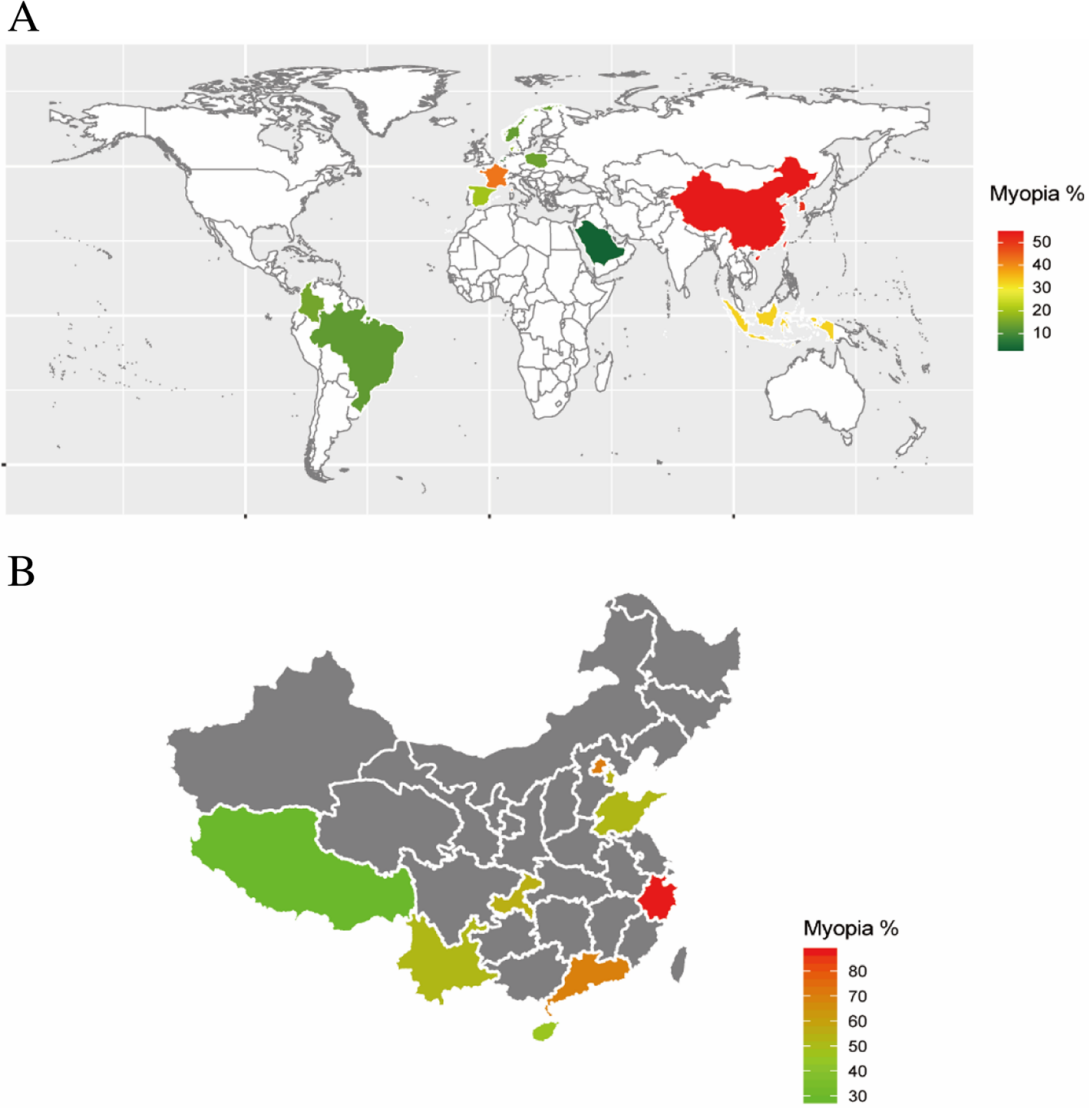
Map of World **a** and China **b** showing the data of myopia prevalence from other studies published in recent 5years, matched on myopia definition and age

Therefore, compared with recently published 5year child and adolescent myopia data from other countries, the prevalence of myopia in our sample was significantly higher than Netherlands (2.4%) [9], Saudi Arabia (2.**7**%) [13], Norway (13.0%) [7], Colombia (14.9%) [14], North India (21.1%) [6], Denmark (17.9%) [15], Spain (20.0%) [11], and Poland (16.33%) [16]. However, it is similar to France (42.7%) [17], but lower than in Korea (51.9%) [18] (see Table 3). Compared with different regions across China, the prevalence of myopia in our study within the same age range of children was lower than that of Feng Hua (87.65%, Eastern China) [19], Guangzhou (69.9%, Southern China) [20], Beijing (70.9%, Northern China) [21], Qingdao (52.02%, Eastern China) [22], Chongqing (54.9%, Western China) [23], and Tianjin (53.9%, Northern China) [24], but higher than Mangshi (35.9%,Western Rural China) [25], and Tibet (28.51%, Plateau of China) [26] (Table 3) (Fig.5).

**Table 3:** Summary of myopia prevalence (%) from this study and from other studies published in recent 5years, matched on myopia definition and best matched on age

Country	Age (years)	n	Myopia% (-0.50D)	High Myopia (-6.00D)	Hyperopia% (+0.50 D)	Latitude	Cycl-oplegia
Norway	1619	393	13	0.5	57	60.4 N	YES
North India	912	516	27	NA	NA	16.4 N	YES
Brazilian	911	266	6.4	NA	67.1	22.9 S	YES
	1315	167	12.6		59.8		
Poland	913	4875	14.01	NA	NA	52.1 N	YES
Denmark	9.715.4	307	17.9	NA	NA	55.8 N	YES
France	1019	8289	42.7	1.8	NA	48.7 N	NO
Korea	519	7486	51.9	5.0	13.4	37.3 N	NO
Netherlands	6	5711	2.4	NA	NA	53.2 N	YES
Saudi Arabia	310	1,893	2.7	NA	1.5	24.3 N	NO
Colombia	15	NA	14.7	NA	32.3	5.1 N	NO
Spain	57	6152	20	3.6	NA	43.4 N	NO
Indonesia	812	410	32.68	8.54	0.73	6.1 S	NO
Eastern China	18	43,858	87.65	16.6	NA	29.4 N	NO
Eastern China	1015	4890	52.02	5.7	NA	36.0 N	YES
Northern China	618	35,745	70.9	19.4	NA	40.2 N	NO
Southern China	7.212.2	1669	69.9	3.1	NA	23.2 N	NO
Western China	615	1858	54.9	2.42	3.0	29.0N	YES
Northern China	612	527	53.9	NA	NA	38 N	YES
Eastern China	519	4801	63.1	9.4	NA	29.02 N	NO
Our study	615	31,524	45.97	0.96	13.80	20 N	NO

The overall prevalence of high myopia was very low (1.0%), lower than many studies with a similar age group, such as studies from Korea (5%) [18], Spain (3.6%) [11], Indonesia (8.54%) [27], and the aforementioned Chinese cities. The overall prevalence of hyperopia (SE+0.50 D) (13.8%) is significantly higher than Indonesia(0.73%) [27], America(5%) [28], and Chongqing (3%, Western China) [23]; Significantly lower than Norway (57.0%) [7], Brazil (59.8%) [29], and Colombia (32.3%) [14]. However, it is similar to Korea (13.4%) [18]. A thorough comparison of refractive error based on studies published during the last 5years is summarized in Table 3.

Mean SE in our study was better than Guangzhou (-1.71.9 D) [20], Tianjin (-0.991.69 D) [24], in 612year-olds, and Yiwu (-2.612.01D) [30], in 12year-olds. It was however worse than Poland (+0.551.23D) [16], and Norway (+0.511.29D) [7]. In this study, the trends of different SE were also calculated. The trends showed a low ratio of SE-5.00 and SE-6.00. This indicated another feature that although myopia was common, it could develop into high myopia slowly (Fig.5).

The exact mechanism associated with myopia was undetermined, but could be explained by many related factors, such as genetic factors, environment, lower refractive status at baseline, shorter reading distance, outdoor exposure and so on. Overall, increased incidence of myopia remains a global public health challenge, which necessitates novel therapeutic methods to curb its progression.

Even though genetic factors are considered to be important in myopia development, especially high myopia [9, 31], a multitude of studies on the large increase in incidence propose a much stronger effect of environmental factors in younger students. Some researchers hold the view that intensive continuing education and limited time outdoors are major risk factors [32]. However, little evidence of the relationship between time spent at work and myopia was reported in Norway [7]. This is important as Norway has a low myopia prevalence and being outdoors is a part of growing up. Many of the studies on myopia suggest that longer outdoor light exposure time correlated with a significant reduction in myopia prevalence and incidence among school students [11]. High levels of daylight exposure were considered to be the environmental factor of greatest importance in preventing myopia [33]. Haikou City, as the capital of Hainan Province, located in low-latitude tropical regions in China, experiences Chinas longest hours of sunshine and great radiant energy. The average sunshine hours are more than 2000h per year. The hypothesis that daylight exposure explains why prevalence of myopia in our study was lower than other regions of China cannot explain why it was higher than other countries at the same latitude, such as North India [6]. In addition, at the same latitude, the incidence rate of myopia was higher than in other Asian children of the same age, but with lower high myopia prevalence [18]. Therefore, daylight exposure may be an influential factor, but it does not fully explain myopia development.

Results of another study showed that increased computer use is related to myopia development before children reach 10years of age. Outdoor exposure may be important for intervention against myopia because it could mitigate near work activities, including computer use, reading time and distance [34]. A systematic review of several studies has indicated an association between screen time and myopia, but Meta-analysis suggested that screen time was not related with prevalent and incident myopia. So, there is still a debate whether digital screen time would induce the higher risk of myopia [35]. However, there is no question that more near work and less time spent outdoors would be affected by the increased use of digital devices.

As mentioned in the literature review, previous studies have noted the importance of UVB exposure in myopia. Increased UVB exposure reduces myopia, especially in adolescence [36]. Violet light suppressed the axial length elongation in the chick myopia model, and myopia suppressive gene EGR1 was upregulated as revealed by expression microarray analyses [37]. Several reports have shown that the increasing quartile of total UVB can contribute to decreased prevalence of myopia [38].

Hainan, with the longer time of UV exposure, is the smallest and southernmost province of China. As compared to many studies conducted in other provincial capital cities of China, low overall myopia prevalence (46.0%) and low overall high myopia prevalence (1.0%) were found in our screening population. In addition, numbers of nonmyopic students newly developing myopia annually are lower. The possible reasons for this difference may be associated with environmental influences. It is likely that the UVB exposure is one of the important factors in myopia development.

Our study contributes new knowledge to the field attributed to the latest and large population-based screening methods. When we defined refractive errors, monocular myopia, hyperopia and astigmatism were taken into consideration rather than performing statistical analyses on one of two eyes. Our approach thus represents the overall data unlike reports in other published papers. Our calculated myopia prevalence would therefore be artificially lower than now reported if we simulated the alternative approach from such studies.

Although myopia was defined as combined spherical equivalent with normal UCVA to reduce over-measuring of myopic magnitude, there is still a notable limitation of the present study. There is lack of cycloplegic refraction that might lead to over-estimation of myopia and under-estimation of hyperopia due to accommodation. The difference in refractive error prior to, and after cycloplegia was about 0.37 diopters in a small sample; consistent with other research findings [23]. Regardless, the difference between pre- and post-cycloplegia was small and may not impose a significant clinical influence under real-world conditions in a large sample population. We also need further investigations to acquire data on ocular biometry and more detailed information about students. We certainly aim for this to be our next research focus.

Genetic and environmental risk factors may be taken into consideration to explain how refractive errors develop differently. Our results presented a higher myopia prevalence than European countries, which indicated the potential presence of a genetic predisposition to myopia in Asian populations. All in all, myopia in Asians is a serious health problem. Genetic heterogeneity, variation in circannual adaptation and environmental factors including timing and behavior patterns of exposure to myopia-generation are related to greater shift towards myopia. Therefore, it is important to dedicate effective control methods to slow myopia progression.

## Conclusions

Myopia was the most common refractive error in southernmost province in China (Hainan). Its prevalence increased with age, while hyperopia prevalence showed a decreasing trend. However, myopia, especially high myopia prevalence was much lower than in other urban regions across China, as residents of Hainan may benefit from more ultraviolet B (UVB) radiation during daylight exposure.

## Data Availability

The data used to support the findings of this study are available from the corresponding author upon request.

## Abbreviations

VA: Visual acuity
UCVA: Uncorrected visual acuity
UVB: Ultraviolet B
RE: Refractive errors
SE: Spherical equivalent
